# Novel circRNA discovery in sheep shows evidence of high backsplice junction conservation

**DOI:** 10.1038/s41598-020-79781-2

**Published:** 2021-01-11

**Authors:** Endika Varela-Martínez, Giulia I. Corsi, Christian Anthon, Jan Gorodkin, Begoña M. Jugo

**Affiliations:** 1 Department of Genetics, Physical Anthropology and Animal Physiology, Faculty of Science and Technology, University of the Basque Country (UPV/EHU), Bº Sarriena, 48940 Leioa, Spain; 2 Department of Veterinary and Animal Sciences, Center for Non-Coding RNA in Technology and Health, University of Copenhagen, Thorvaldsensvej 57, 1871 Frederiksberg, Denmark

**Keywords:** Computational biology and bioinformatics, Genetics, Immunology

## Abstract

Circular RNAs (circRNAs) are covalently closed circular non-coding RNAs. Due to their structure, circRNAs are more stable and have longer half-lives than linear RNAs making them good candidates for disease biomarkers. Despite the scientific relevance of these molecules, the study of circRNAs in non-model organisms is still in its infancy. Here, we analyse total RNA-seq data to identify circRNAs in sheep from peripheral blood mononuclear cells (PBMCs) and parietal lobe cortex. Out of 2510 and 3403 circRNAs detected in parietal lobe cortex and in PBMCs, a total of 1379 novel circRNAs were discovered. Remarkably, around 63% of all detected circRNAs were found to be completely homologous to a circRNA annotated in human. Functional enrichment analysis was conducted for both tissues based on GO terms and KEGG pathways. The enriched terms suggest an important role of circRNAs from encephalon in synaptic functions and the involvement of circRNAs from PBMCs in basic immune system functions. In addition to this, we investigated the role of circRNAs in repetitive vaccination experiments via differential expression analysis and did not detect any significant relationship. At last, our results support both the miRNA sponge and the miRNA shuttle functions of CDR1-AS in sheep brain. To our knowledge, this is the first study on circRNA annotation in sheep PBMCs or parietal lobe cortex samples.

## Introduction

Circular RNAs (circRNAs) are a new class of covalently closed circular non-coding RNAs, formed when a splice donor and upstream acceptor from a linear RNA are linked together, a process also called backsplicing^1^. Due to their circular structure, circRNAs are more stable, resistant to RNAse R and have longer half-lives than linear RNAs^2^, making them good candidates for disease biomarkers. Despite being discovered long ago, with the first circular molecules (viroids) revealed by electron microscopy in 1976^3^ and the first endogenous circRNA originating from the *DCC* tumour suppressor reported in humans in 1991^4^, for a long time circRNAs were thought to be low abundance products derived from splicing errors^5^. With the recent increase in high-throughput sequencing studies, it was shown that these molecules are more common than initially thought and that some of them have important roles in multiple pathways^6,7^. The exact mechanism of circularization is not totally understood, but multiple factors have been related. It has been shown that circRNA biogenesis is positively correlated by RNA polymerase II elongation rate^8^. In addition, multiple reports have shown that reverse complementary sequences in the flanking introns of the backspliced exons brings under close proximity the splice sites^9^, allowing for the canonical spliceosomal machinery to be employed. Furthermore, RNA binding proteins such as Quaking (QKI), muscleblind (MBL) and fused in sarcoma (FUS) have also been reported to promote circRNA biogenesis^9^.

Although the biological function of most circRNAs remains unknown, some circRNAs have been shown to contain clusters of miRNA binding sites that function as miRNA sponges (e.g., the circRNAs related to CDR1 and SRY sequester miR-7 and miR-138, respectively)^10^. Thus, circRNAs may interfere in the usual miRNA-mRNA binding procedures. Other circRNAs have been shown to contain sequences that can act as internal ribosome entry sites (IRESes), such as circ-ZNF609^11^, thus can potentially code for proteins. However, their actual translation in vivo remains to be probed. Last, circRNAs can regulate a number of processes via protein-binding activity (e.g., the circ-FOXO3 forms a ternary complex with p21 and CDK2)^12^.

Recent reports have associated circRNA expression with multiple diseases and it has opened a new field for diagnosis and treatment. It has been shown that circRNA levels increase with age in brain, but the same has been shown in age-associated neurological disorders such as Alzheimer’s disease and Parkinson’s disease^13^. In addition to evidence of circRNAs playing a role in diseases such as atherosclerotic vascular disease risk, osteoarthritis and diabetes, it has been shown dysregulated expression of circRNAs in multiple types of cancer, including colorectal cancer, hepatocellular carcinoma and breast cancer, among others^14^.

More recently, many circRNAs have been reported to be expressed abnormally and play important roles in the progression of autoimmune diseases such as rheumatoid arthritis, systemic lupus erythematosus or multiple sclerosis^15^. Thus, circRNAs may not only serve as potential biomarkers but also act as immune regulators an offer potential opportunities for therapy.

Non-living vaccine antigens, especially purified or recombinant subunit vaccines, are often poorly immunogenic and require additional components to help stimulate protective immunity based on antibodies and effector T cell functions. These additional components, termed adjuvants, are added to vaccines to achieve a better protection, with the aluminium-based ones (especially aluminium hydroxide) being some of the most widely employed adjuvants in human and animal vaccines. Despite its widespread use and its probed safety record, the adjuvant’s mechanism of action is not fully understood.

Recently, some concerns regarding the safety of aluminium adjuvants has been raised, due to the possibility for aluminium adjuvants to reach distant organs such as spleen or brain after a long-term exposition. It was shown that after intramuscular injection of the aluminium adjuvant in mice, the material was translocated at a very slow rate in normal conditions to draining lymph nodes (DNL) and thereafter was detected as associated with phagocytes in blood and spleen^16^. In addition, several studies have addressed the translocation of aluminium to the brain^16–18^. However, this remains a subject with much controversy in the scientific community and there is no complete agreement regarding the translocation and biopersistence of this material^17,19,20^.

In sheep, a form of the autoimmune/autoinflammatory syndrome induced by aluminium-adjuvants has been described as linked to repetitive inoculation with aluminium-containing vaccines^21^. In this species, a number of circRNAs were previously identified from RNA sequencing data. Li et al. detected 6133 and 10,226 circRNAs in prenatal and postnatal muscle and pituitary glands of sheep, respectively^13,14^. Interestingly, they observed an association of some circRNAs with economically important traits, such as the growth and development of muscle related signaling pathways in the first tissue and the regulation of hormone secretion in the second. In addition to this, the same group identified 9231 circRNAs differentially expressed in the estrus and anestrus pituitary system of sheep^15^. Last, 886 circRNAs were detected in the skeletal muscle by Cao et al., and some of them were reported to be involved in muscle cell development and signaling pathway^16^. Characterizing the circRNA profiles of specific tissues and cell types is a promising way to reveal functional properties of circRNAs.

Until now, there has been no study trying to address the functional role of circRNAs in aluminium adjuvancy through total RNA sequencing data analysis, nor attempts of annotating circRNAs in sheep peripheral blood mononuclear cells (PBMCs) or parietal lobe cortex samples. In this work the circRNAs of these two tissues will be characterized and their expression in animals with different adjuvancy treatments assessed. Characterizing how circRNAs are expressed in different tissues can improve our understanding of the sheep transcriptome and analysing their expression in vaccinated or adjuvanted animals could add information on the role of circRNAs in the immune response to aluminium adjuvants.

## Results

### CircRNAs characterization and distribution in encephalon and PBMCs

**Figure 1:**
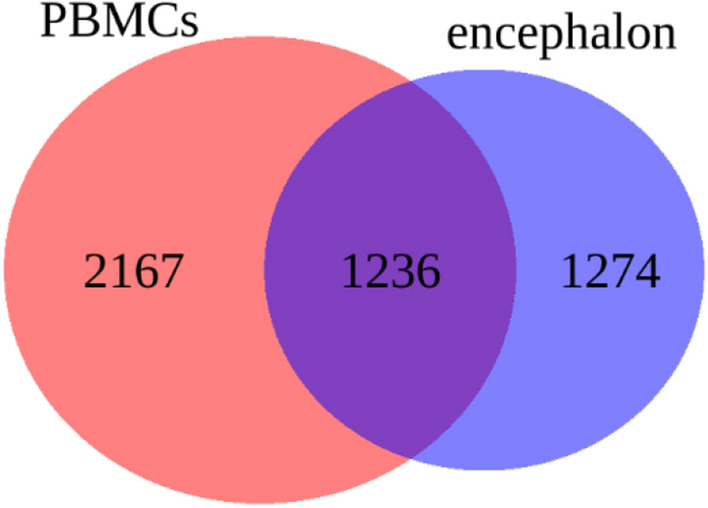
Venn diagram with the number of circRNAs detected in each tissue after filtering for a minimum expression in at least three samples.

Total RNA-seq data was produced from RNA samples extracted from encephalon and PBMCs. The data have been previously used for in depth differential expression analyses^22,23^ and it has been re-analysed for circRNA annotation. Two bioinformatics tools, Segemehl^24^ and DCC^25^, were selected for circRNA identification, which resulted in 12,475 and 60,375 candidate circRNAs in encephalon and 19,611 and 63,138 candidate circRNAs in PBMC samples by segemehl and DCC, respectively. Out of all the circRNAs detected in the encephalon, 4996 had concordant coordinates in both tools. After filtering circRNAs based on their abundance and expression patterns among samples (see “Material and methods”), 2510 circRNAs were selected for subsequent analyses. In PBMCs, 10,414 circRNAs were concordant between tools. After filtering, 3403 circRNAs were retained for further analysis. Details about filtered circRNAs are available as Supplementary Data S1 and S2 for encephalon and PBMCs, respectively. The naming of circRNAs in each tissue list was performed by assigning sequential unique numeric identifiers. From the 2510 and 3403 circRNAs detected in encephalon and PBMCs, 1236 were present concordantly in both tissues (Fig. 1). The counts from DCC were taken as reference abundance values.

In the available literature a number of studies have described the principal characteristics of circRNAs in human and mouse^10,26^. In our sheep data, in both tissues, we observe that the longer the chromosome, the more circRNAs are detected (Supplementary Fig. S1), and that the circRNAs are most commonly formed by two or three exons, being those composed of two exons the most prevalent ones (Supplementary Fig. S1). This is in accordance with what was previously described in other species^10^. A representation of the location of each circRNA in the reference genome is given in Supplementary Fig. S2 for encephalon and Supplementary Fig. S3 for PBMCs.

Out of the 2510 candidate circRNAs detected in encephalon, 2372 overlap with 1642 annotated sheep genes. Of those circRNAs that originated from an annotated gene, 1927 were concordant with an annotated exon–intron boundary in both ends, while in the other cases, despite the overlap with an annotated gene, at least one end was not concordant with an annotated exon–intron boundary. Concerning the 3403 circRNAs detected in PBMCs, 3249 were found to originate from 2006 annotated sheep genes. Of these, 2597 were concordant with an annotated exon–intron boundary in both ends. In some cases, the cause of the discrepancy between the annotated exon–intron boundaries and the circRNA backspliced junctions could be explained by the incomplete state of the sheep gene annotation. The majority of genes host only one circRNA in both tissues (Supplementary Fig. S1).

### Sheep circRNAs are conserved

**Figure 2:**
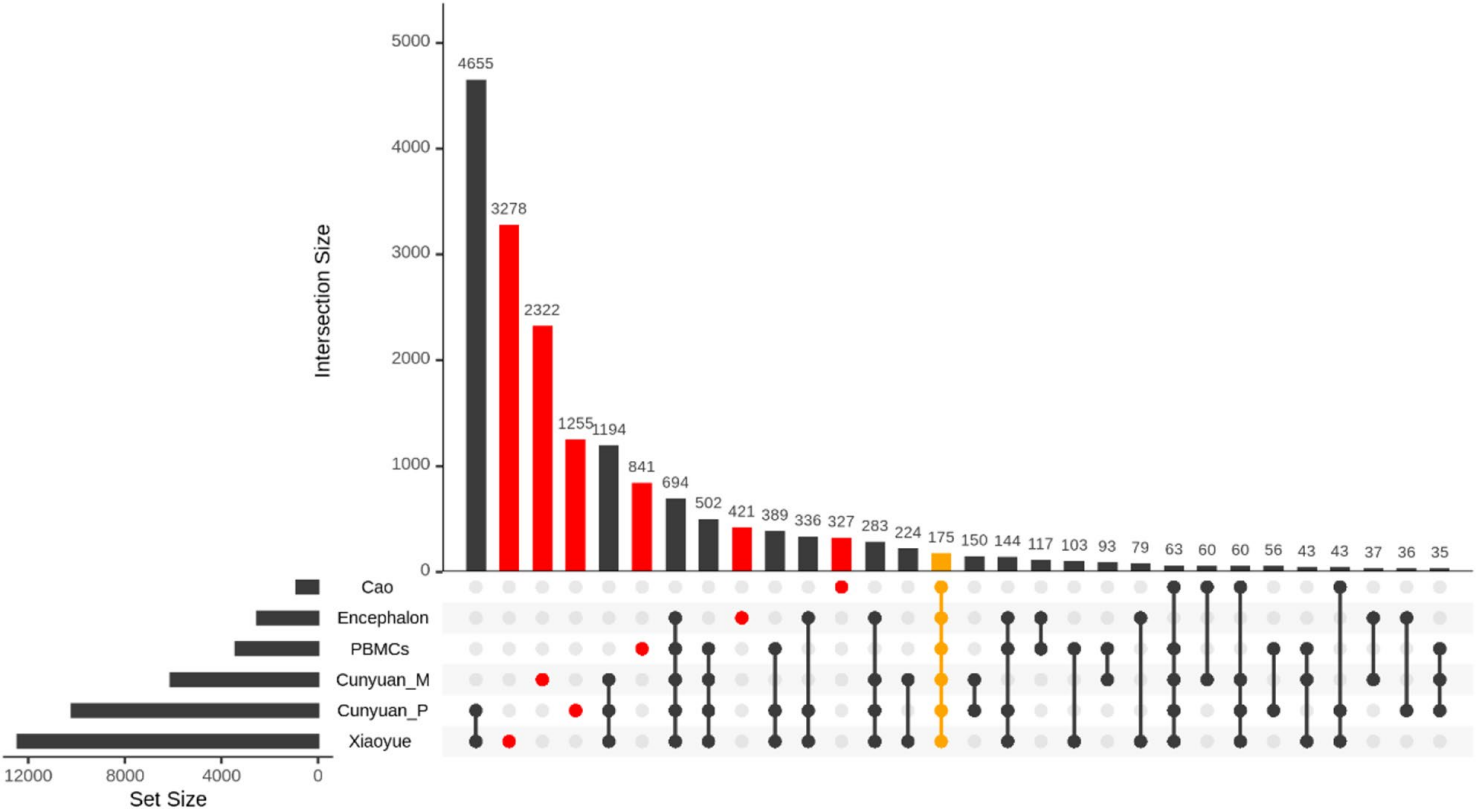
UpSet plot with the comparison of detected circRNAs in different studies. Encephalon and PBMCs refers to the circRNAs detected in this study, while Cunyuan_P (pituitary gland), Xiaoyue (pituitary gland), Cunyuan_M (longissimus dorsi muscle) and Cao (longissimus dorsi muscle) refers to the circRNAs detected in^45–48^, respectively. Cells filled with a dot indicate the circRNA is in the corresponding database, while empty cells indicate that the circRNA is not present in the corresponding database. In red the circRNAs that are exclusively expressed in one database and in orange the circRNAs common to all databases. Intersections with less than 30 elements were removed for visualization purposes.

CircRNAs have been shown to be tissue specific and to be evolutionary conserved^27^. The circRNAs detected in this study were compared to others previously identified in other tissues (pituitary gland and longissimus dorsi muscle) in sheep. Notably, only 175 circRNAs were consistently detected in all tissues, including ours (Fig. 2). Such low concordance is in agreement with other studies, which showed that the expression of circRNAs is tissue-dependent^8^. In addition, our results showed that 421 and 841 circRNAs were exclusive to the encephalon and PBMCs data, respectively, while the overlap between the two sets is composed of 117 circRNAs (Fig. 2).

**Figure 3:**
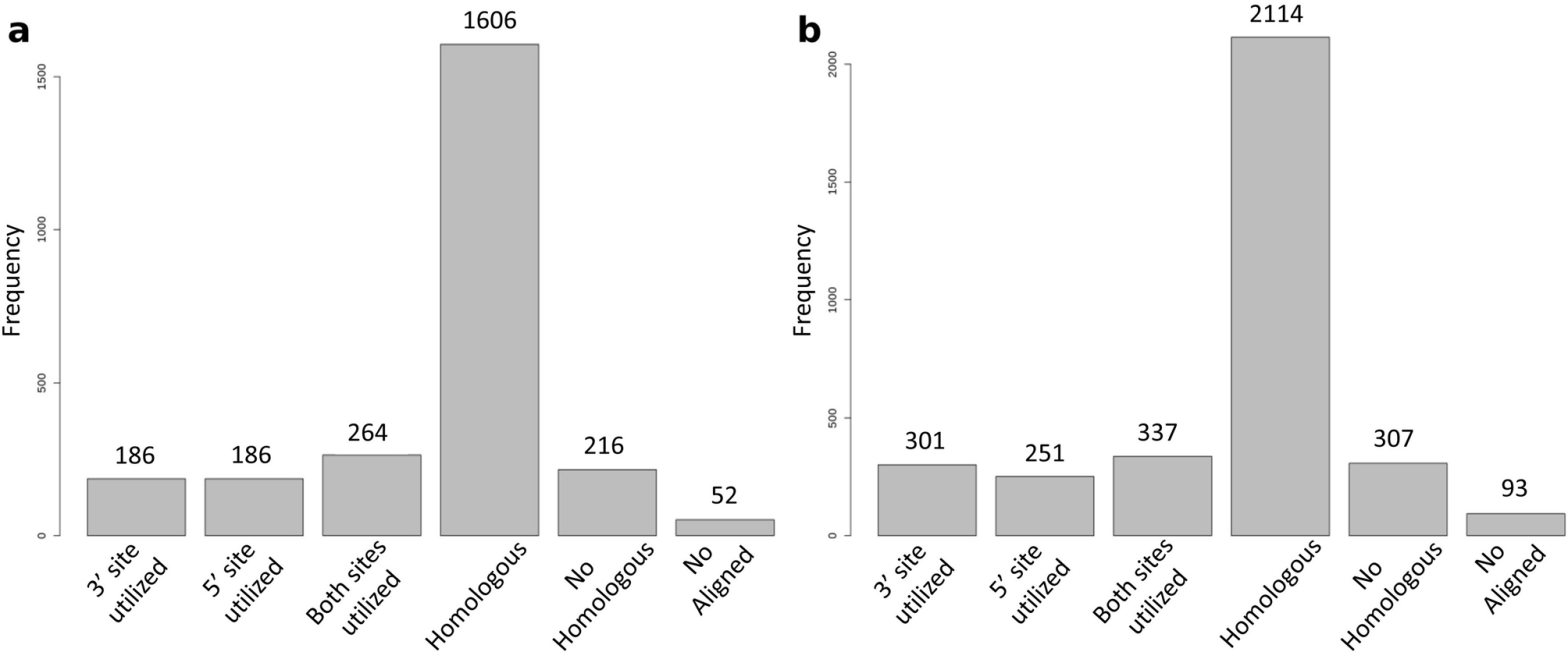
Bar plot with the result of the conservation analysis. In the x-axis the different categories described in “Material and methods” and in the y-axis the number of circRNAs in each category. (**a**) Encephalon; (**b**) PBMCs.

In addition to this, the detected circRNAs were compared to the human circRNAs annotated in CIRCpedia^28^. First, sheep circRNA coordinates were translated to human ones with the UCSC liftOver tool^29^ and classified based on their backsplice junction conservation. Out of the 2510 detected circRNAs in encephalon, 52 splice sites coordinates could not be lifted. For the rest, nearly all had at least one reported human circRNA utilizing one of the splice sites. A total of 1606 (63.98%) circRNAs were completely homologous to a human circRNA (Fig. 3a). In PBMCs, out of the 3403 detected circRNAs, 93 splice sites coordinates were not lifted to human, while 2114 (62.12%) circRNAs were found to be completely homologous to a human circRNA (Fig. 3b).

Given that circRNAs include exons of coding genes, sheep circRNAs completely homologous to a human one but lacking a gene annotation in sheep were also screened for possible corresponding genes annotated in human (Supplementary Table S1).

### Enrichment analysis

**Figure 4:**
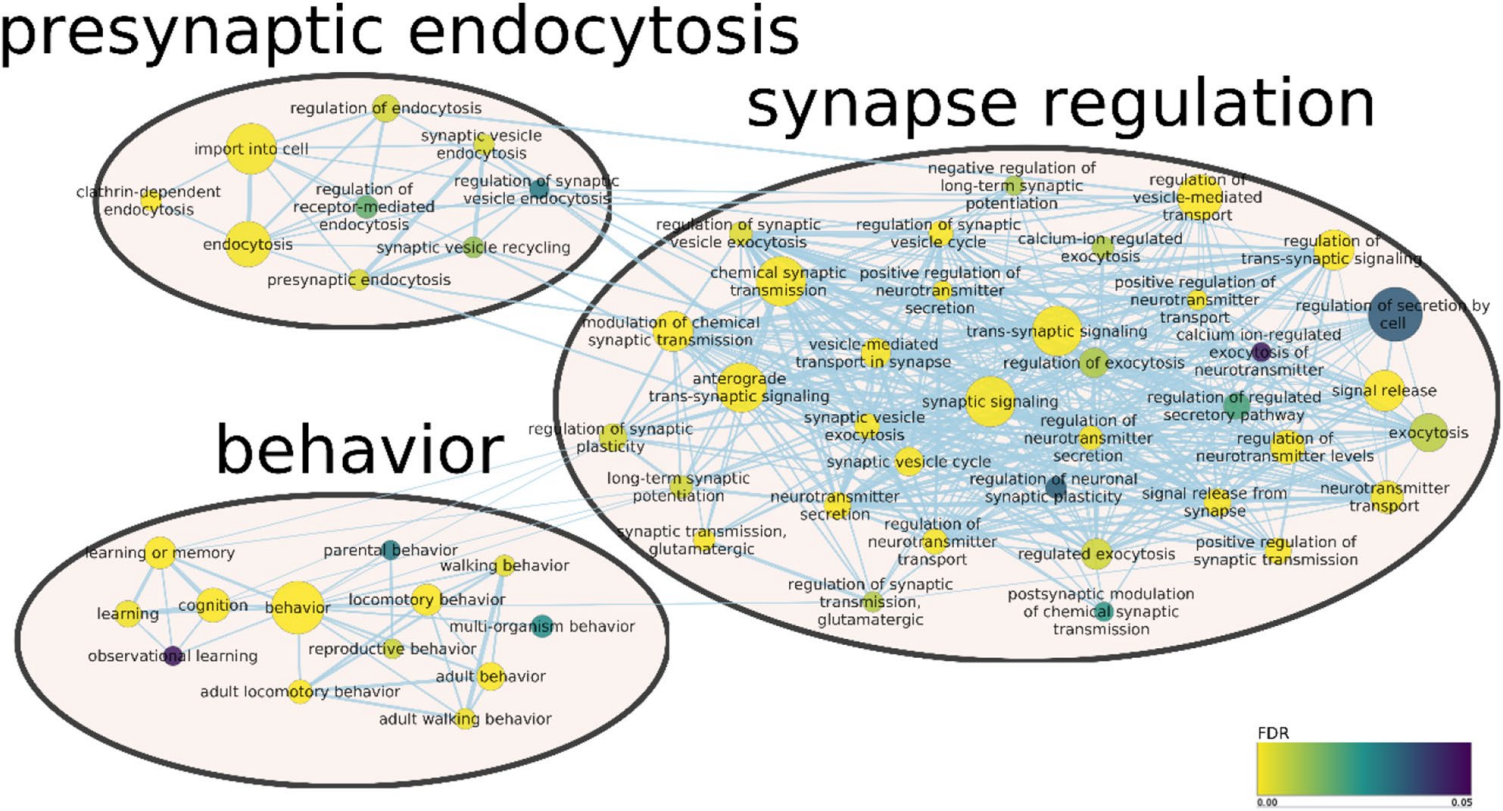
Sub-network from enriched GO terms by g:Profiler in encephalon and visualized in Cytoscape after clustering with Autoannotate. Node size correspond to number of genes expressed from the term; edge size represents the number of genes that overlap between different terms; node colour represents the significance level (FDR).

**Figure 5:**
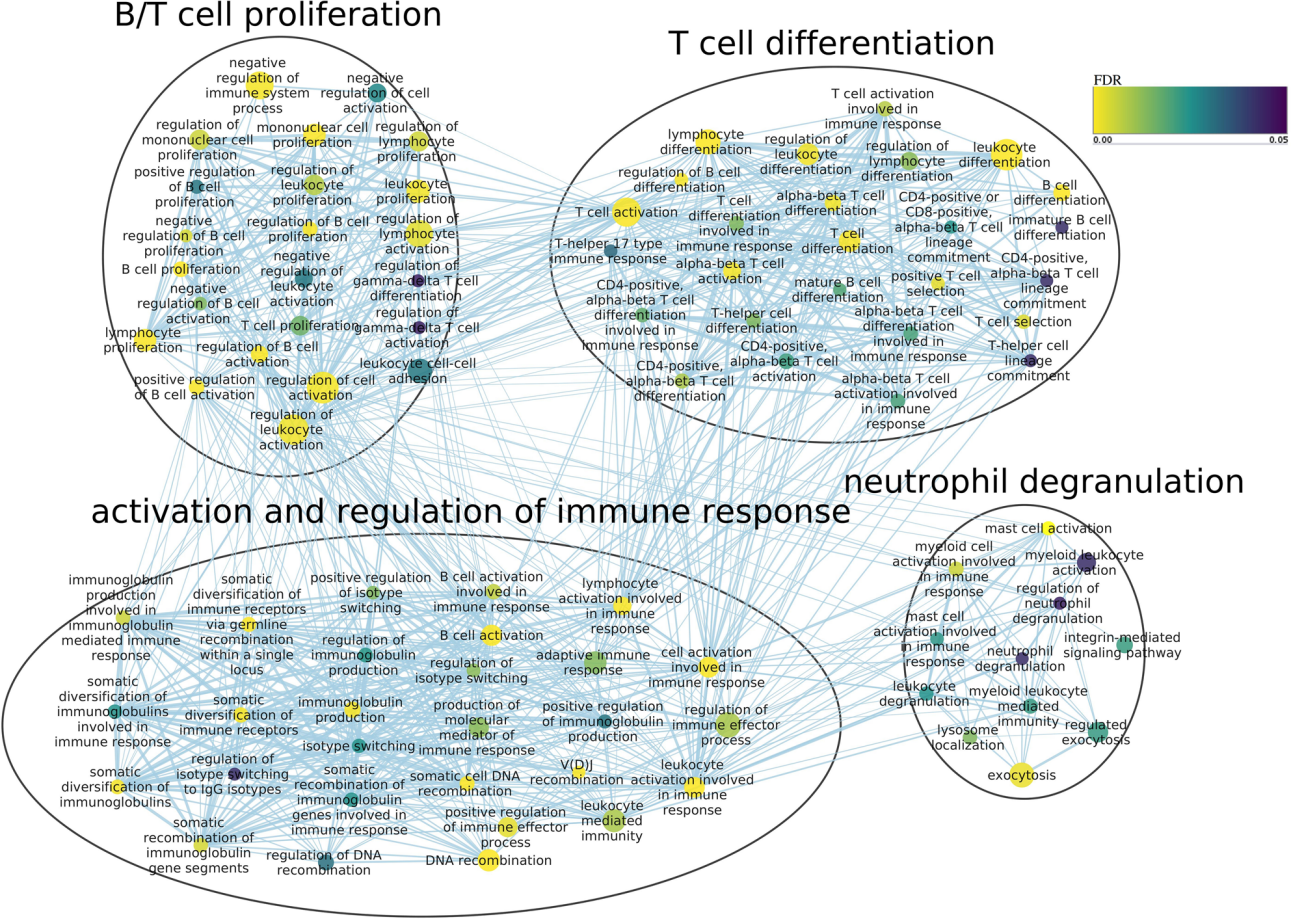
Sub-network from enriched GO terms by g:Profiler in PBMCs and visualized in Cytoscape after clustering with Autoannotate. Node size correspond to number of genes expressed from the term; edge size represents the number of genes that overlap between different terms; node colour represents the significance level (FDR).

**Figure 6:**
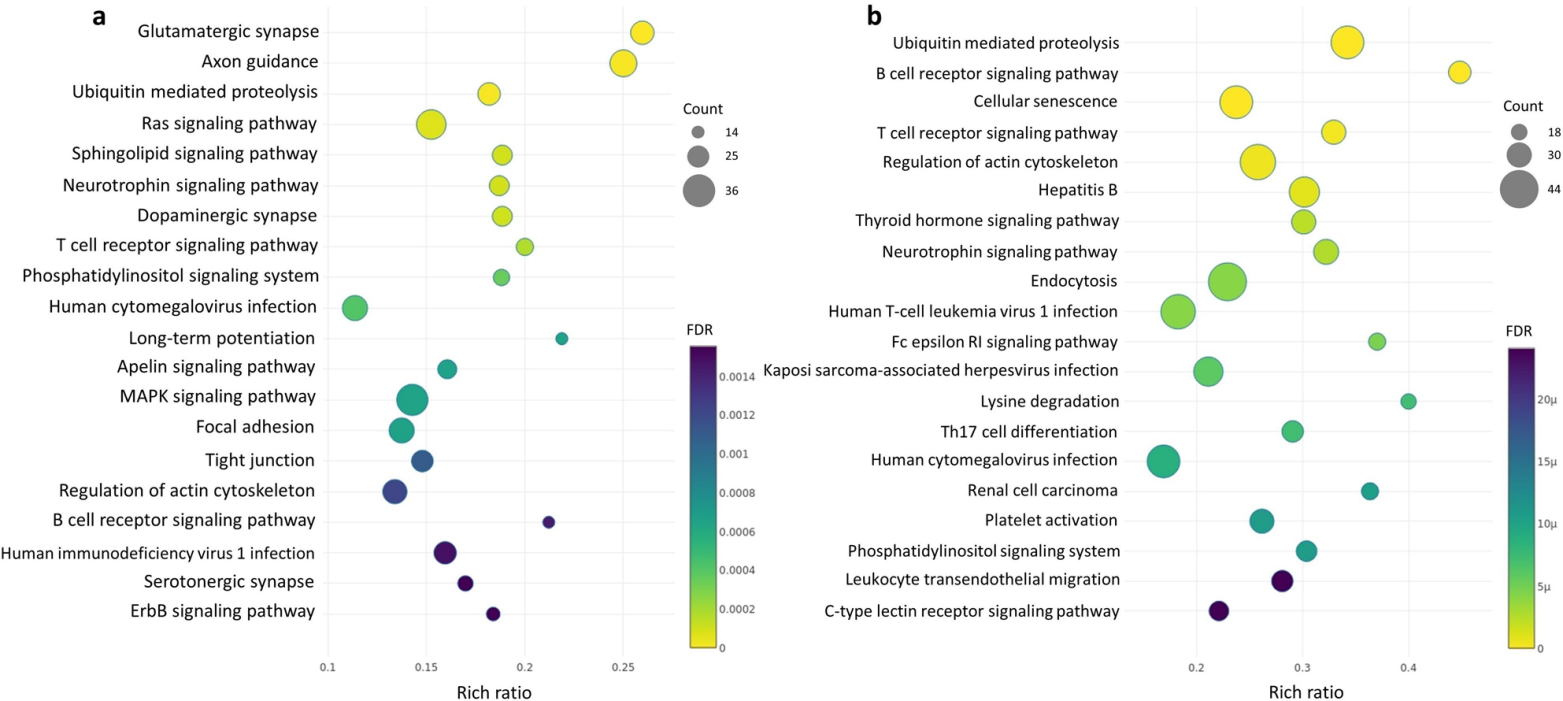
The 20 most enriched KEGG pathways by g:Profiler. The bubble plots show in the Y-axis the enriched KEGG pathways, while in the X-axis the rich ratio is represented (rich ratio = amount of differentially expressed genes in the term/all genes included in the term). Size and colour of the bubble represent the number of differentially expressed genes in the KEGG pathway and enrichment significance (FDR), respectively. (**a**) Encephalon; (**b**) PBMCs.

A functional enrichment analysis was conducted with g:Profiler^30^ on the GO^31^ and KEGG^32^ databases for both tissues, by considering the terms annotated for the parental genes of the detected circRNAs and after setting as background all the genes expressed in the corresponding tissue. Terms with an FDR less than 0.05 were selected as significant. The enriched GO terms are represented as networks in Supplementary Fig. S4 and S5. Selected highly connected sub-networks of interest are shown in Figs. 4 and 5. The 20 most enriched KEGG pathways are shown in Fig. 6a,b, for encephalon and PBMCs, respectively. Among the GO terms significantly enriched in encephalon, there are a number of terms related to synapse regulation, presynaptic endocytosis, behaviour, brain development and myelination, while among the KEGG pathways glutamatergic synapse, dopaminergic synapse and serotonergic synapse were enriched, suggesting an important role for some circRNAs in synaptic functions. Instead, in PBMCs, we retrieved GO terms related to B- and T-cell proliferation, T-cell differentiation, activation and regulation of immune response and neutrophil degranulation. In both tissues, the KEGG T-cell receptor signaling pathway and B-cell receptor signaling pathway were enriched, suggesting that some circRNAs may be involved in basic immune system functions.

### circRNAs acting as sponges

To identify circRNAs which could function as miRNA sponges, we compared all 2510 (encephalon) and 3403 (PBMCs) predicted circRNAs with clusters of miRNA binding sites reported by Pan et al.^33^ in the human genome, a dataset that comprises a total of 3673 predicted sponges for 1250 miRNAs. Out of 3 (encephalon) and 4 (PBMCs) sheep circRNAs overlapping one or more candidate sponges-miRNA pairs, we filtered out those entries for which the predicted sponged miRNA does not have a homologous pre-miRNA in sheep. As a result, in the encephalon tissue we identified 1 circRNA (circRNA4960) overlapping predicted sponges for two miRNAs (miR-7 and miR-1224), while in PBMCs we retained two circRNAs, circRNA2342, which overlaps predicted sponges for miR-409, miR-383, miR-370, miR-369 and miR-212, and circRNA8181 for miR-124 (Supplementary Table S2). Then circRNA-target-miRNA pairs were screened for miRNA binding sites in both human and sheep circRNA sequences with RIsearch2^34^. After removing overlapping binding sites as described in Pan et al*.*^33^*,* 44 and 65 binding sites were respectively found on circRNA4960 for miR-7 and miR-1224. Although the sheep circRNA4960 is shorter than the corresponding cluster of miRNA binding sites detected in human for miR-7 and miR-1224, the per-base binding sites ratio is higher in sheep, further underlying a possible functional role of this molecule in the sheep brain.

One of the most well characterized circRNAs in brain is the one related to the CDR1 gene^35^. Although CDR1 is not annotated in sheep, blasting the human sequence of this gene against the sheep reference genome results in a single hit, matching a region of circRNA4960, detected in our encephalon samples. We lifted the coordinates of the sheep backsplice junctions (sheep genome version Oar_3.1) to the human genome (version hg38) with the UCSC liftOver tool^29^ and found that circRNA4960 is homologous to the human CDR1-AS. Interestingly, circRNA4960 was one of the most expressed in our cortex samples (Supplementary Table S3). Among the highly expressed circRNAs detected in encephalon other two were homologous to previously characterized human circRNAs, circRNA4266 and circRNA4357, which originate from HOMER1 and ZNF609 genes, respectively.

In addition, recent studies have shown that miR-671 has sufficient complementarity with CDR1-AS^36^. Interestingly, the binding pattern of miR-671 in sheep is identical to the human one and includes 13 canonical base pairs in the seed region, and only 1 mismatch over the entire sequence (Supplementary Fig. S6). Hence, our results support both the miRNA sponge and the miRNA shuttle functions previously proposed for CDR1-AS in brain and suggest a possible similar mechanism for miR-1224, which is reported as highly expressed in brain according to the Genotype-Tissue Expression (GTEx) Project v8.

The screening of circRNA-target-miRNA pairs identified in PBMCs showed that miRNA binding sites are scattered far away from one another over both the exonic and the much longer intronic regions of circRNA2342 and circRNA8181, with few bindings overlapping with the clusters of miRNA binding sites identified in human, hence we could not infer any sponge activity for these circRNAs. The complete list of binding sites identified for sheep circRNA-miRNA pairs in both encephalon and PBMCs candidate circRNA sponges is available in Supplementary Table S2.

### No differential expression due to repetitive vaccination

Our preliminary analysis of transcripts expression showed that the adjuvant sample 116-E derived from the encephalon tissue was an outlier, thus it was removed prior differential expression analysis. A PCA showing clusters of samples is shown in Supplementary Fig. S7. Differential expression analysis was performed with the R package DESeq2^37^. We did not detect any differentially expressed circRNA in any comparison after considering an adjusted p-value < 0.05 as cut-off. We also performed differential expression analysis normalizing the data as spliced reads per billion mapping (SRPBM), and by applying a Kruskal–Wallis test before correcting for multiple comparison with the Benjamini and Hochberg method. Also in this case, there were no significant differences between groups when an adjusted p-value < 0.05 was taken as cut-off.

For the PBMCs samples, the Harman R package^38^ was applied to remove any batch effect in the data after normalizing by SRPBM. The PCA with the corrected data is shown in Supplementary Fig. S7. Then, both the limma package^39^ and Kruskal–Wallis test were used to test for differential expression, but no circRNA was found to be differentially expressed in any comparison with an adjusted p-value < 0.05.

## Discussion

CircRNAs are a novel class of endogenous non-coding RNAs with a cyclic structure formed through a covalent bind of a linear transcript. Lately, circRNAs have gained more attention due to their abundance, their expression levels in specific tissues and their involvement in different biological functions, particularly studied in human and mouse^40–42^. However, studies on circRNAs in non-model organism such as sheep are still lacking, and there is no database recording such data yet. Here, we improved the annotation of circRNAs in sheep by adding a total of 1379 novel circRNAs, combined with relevant information such as conservation and potential function. This set of robust circRNAs was selected from 2510 and 3403 circRNAs respectively detected in parietal lobe cortex and in PBMCs via in silico analysis of ribo-minus total RNA sequencing data. Most of the identified circRNAs in both tissues are from annotated genes, generally formed by two or three distinct exons, in agreement with what has been previously reported in human and mouse data^43^. In addition, we observe that circRNAs are widely expressed in both of these tissues in sheep, which was somewhat expected since circRNAs are enriched in mammalian brain and human PBMCs^44^.

Some circRNAs have a tissue-dependent or developmental stage-dependent expression pattern^8^. The circRNAs detected in this study were compared to other sheep circRNA identified in pituitary gland^45,46^ and in longissimus dorsi muscle^47,48^. Only 175 circRNAs were detected in all tissues, while several hundreds of circRNAs were exclusive to each tissue. Furthermore, given that numerous circRNAs have exhibited evolutionary conservation between human and mouse^49^, the circRNAs detected in this study were analysed for backsplice site conservation, by comparing them to the human circRNAs available in CIRCpedia. We found that 1606 (63.98%) and 2114 (62.12%) sheep circRNAs have completely conserved backsplice sites between human and sheep in encephalon and PBMCs, respectively. Among the most expressed circRNAs, circRNA4266 and circRNA4357, in order originating from the HOMER1 and ZNF609 genes, had been previously characterized in other species. Consistent with this, it has been shown that the circRNA related to HOMER1 has a regulatory role in cell growth in human bronchial epithelial cells, as its silencing promotes cell proliferation^50^. The circRNA originated from ZNF609 has been shown to adsorb miR-150-5p and to upregulate SP1 transcription factor, promoting the proliferation of nasopharyngeal carcinoma cells^51^. In addition, this circRNA has been related to myoblast proliferation and the fact that its sequence includes an open reading frame and that a fraction of this circRNA is loaded into polysomes indicates that it may encode for proteins^11^.

It was previously proposed that the binding activity between circRNAs and RNA binding proteins (RBPs) can have regulatory effects^52^, which suggests that circRNAs can impact the same functional processes in which the corresponding linear host gene is involved. Under the assumption that the function of a circRNA may be associated with the known function of its parental gene, GO analysis indicated that the circRNAs identified in encephalon are related to synapse regulation, behaviour, learning process and brain development, while KEGG pathway analysis also related these circRNAs to synapses and to pathways implicated in cell proliferation such MAPK/ERK pathways, the last ones being previously linked to circRNAs^43^. In contrast, in the PBMCs samples, GO terms associated with the immune system such as B- and T-cell proliferation, neutrophil degranulation, the MAPK cascade and the NF-κB signaling were enriched, as well as DNA methylation and histone modification, supporting the possibility that circRNAs could be related to epigenetic alterations, as previously suggested^53^. In both tissues the B- and T-cell receptor signalling pathways were enriched, in addition to Fc epsilon RI signaling pathway, Th17 cell differentiation and platelet activation in PBMCs samples, indicating a potential functional role for circRNAs in the immune system response.

Then, we performed a differential expression analysis to find out if circRNAs could have a role in aluminium adjuvancy in vaccines. We did not detect any differentially expressed circRNAs in any of the two tissues, which indicates that circRNAs may not be connected with aluminium adjuvant effects. Despite this, it should be noted that no differential expression analysis software has been specifically designed to handle circRNA data, in which expression levels are generally lower compared to mRNA and are subjected to greater variability.

Moreover, we screened circRNAs for the presence of clusters of miRNA binding sites, following the concept that circRNAs can act as miRNA sponges. We report that the circRNA CDR1-AS, which corresponds to circRNA4960 in this study, contains numerous binding sites for miR-7 and miR-1224, both reported to be expressed in the mammalian brain. In agreement with our expectations, we observed that this circRNA is highly expressed only in our encephalon samples. In addition, recent studies have shown that miR-671 has sufficient complementarity with CDR1-AS to induce AGO2 endonucleolytic cleavage and, based on this, an alternative function for this circRNA molecule as miRNA shuttle system, releasing its miR-7 cargo upon binding with miR-671, has been proposed^36^. It was shown that the binding sites for miR-671 were retained in sheep, supporting its role in cleavage by AGO2.

In conclusion, a number of circRNAs were identified in sheep encephalon and PBMCs samples, expanding our knowledge on the sheep transcriptome. Moreover, several GO terms and KEGG pathways showed that circRNAs may be involved with synapse regulation and cell proliferation in encephalon and with the immune system response and epigenetic modifications in PBMCs. Furthermore, we showed how circRNA functions associated with the presence of clusters of miRNA binding sites are conserved between sheep and human. This study is a first systematic analysis of circRNAs in sheep parietal lobe cortex and PBMC samples, and it is also a first study of the changes in circRNA expression profiles after an aluminium-based adjuvant vaccine inoculation schedule.

## Material and methods

### Ethics statement

All experimental procedures were approved and licensed by the Ethical Committee of the University of Zaragoza (ref: PI15/14). Requirements of the Spanish Policy for Animal Protection (RED53/2013) and the European Union Directive 2010/63 on protection of experimental animals were always fulfilled.

### Datasets

The data samples used in this work have been previously used for in depth differential expression analyses and detailed information about the experimental design and sequencing can be found in the corresponding articles for both tissues, PBMCs^22^ and parietal lobe cortex^23^. Briefly, healthy three-month-old Rasa Aragonesa pure breed lambs from a single pedigree flock, with the condition of not having undergone any kind of vaccination before the experiment, were selected to be placed in the experimental farm of the university of Zaragoza. After a period of two months to acclimatize to the new environment, all lambs were randomly distributed in different treatment groups, each consisting of 7 animals. One of the groups, from now on denominated vaccine group (Vac), received a subcutaneous treatment with commercial vaccines based on aluminium hydroxide adjuvant. Another group, denominated adjuvant group (Adj), received equivalent doses to the commercial vaccines of aluminium hydroxide only (Alhydrogel, CZ Veterinaria, Spain) diluted in phosphate-buffered saline (PBS). Finally, PBS was administered to the control group. Blood samples were taken at the start (before any vaccination) and at the end of the experiment, while for encephalon (parietal lobe cortex) only samples at the end were taken. In Table 1 there is a summary of the samples used for sequencing.

**Table 1:** Sample summary.

Treatment	Time	Samples
**Encephalon**		
Adjuvant	End (Tf)	114-E, 115-E, 116-E, 117-E
Vaccine	End (Tf)	121-E, 122-E, 124-E, 126-E
Control	End (Tf)	131-E, 135-E, 136-E, 137-E
**PBMCs**		
Adjuvant	Start (T0)	121-A, 124-A, 125-A
	End (Tf)	121-B, 124-B, 125-B, 125-B^a^
Vaccine	Start (T0)	111-A, 114-A, 116-A
	End (Tf)	111-B, 114-B, 116-B

^a^Same RNA sample obtained with a conventional TRIzol extraction method.

The complete experiment lasted 475 days, from February 2015 to June 2016. During that period of time, nine different vaccines were administered, which comprises a total of 19 inoculations throughout 16 different inoculation dates. A total amount of 81.29 mg of Al per animal was given in the Vac and Adj groups. A detailed list of the commercial vaccines used in this study can be seen as supplementary material in a previous publication^22^.

Out of all the animals, only 12 (four animals per group) and 6 (three animals per group at the start and at the end of the experiment) were selected for sequencing from encephalon and PBMCs, respectively. For both tissues, Illumina Total RNA-seq libraries were used and sequenced with a high sequencing depth.

### CircRNA identification

First, a read quality filtering and trimming was performed with Trimmomatic^54^ [v0.38] using the following criteria: (1) adaptor removal with the “palindrome” mode for paired-end data; (2) trimming of bases from the start or end of a read if their quality dropped below a Phred value of 20; (3) trimming of reads if the average quality within a sliding window of five nucleotides fell below 20; and (4) read filtering if their length was sorter than 40.

For circRNA identification two tools were selected, segemehl^24^ [v0.3.4] and DCC^25^ [v0.4.7]. Before running segemehl, quality filtered reads were first aligned to the sheep reference genome (Oar_v3.1) with HISAT2^55^ [v2.1.0]. The set of non-aligned reads from the previous step were used to detect circRNAs in segemehl with default parameters. In contrast, for DCC, the quality filtered reads were first aligned to the reference genome with STAR [v2.6.1d]^56^ following DCC author recommendations. Then, the *chimeric.out.junction* files from the previous alignments and a file with repetitive regions in the sheep genome downloaded from the UCSC table browser (RepeatMasker and Simple Repeats tracks) were passed to DCC. DCC was run with default parameters, except that we require a circRNA had to be expressed with one read in at least one sample to be reported. For further analysis, different filtering criteria were tested for the encephalon and PBMC tissues, as they were subjected to different experimental setups. In both tissues circRNAs needed a minimum of 2 read counts to be considered as expressed. In addition, in encephalon, circRNAs were required to be expressed at least in the same three samples in both tools, while in PBMCs they needed to be expressed at least in the same three samples from one group in both tools. The expression counts for the detected circRNAs and host genes were taken as reference from DCC, focusing mainly in exonic circRNAs for further analysis (still referring them as circRNAs throughout the text).

### Conservation analysis

The main databases of circRNA annotation are focused on human, mouse, rat, zebrafish, fly and worm, being sheep circRNA data not submitted to any database to date. A literature search of articles in which circRNAs in sheep are detected and are given at least as supplementary material was done in an attempt to compare the circRNAs annotated in this study. Four studies focusing on two different tissues were found: two from the pituitary gland^45,46^ and another two from the longissimus dorsi muscle^47,48^.

Then, the detected circRNAs were compared to the ones annotated in CIRCpedia^28^ for human. The following steps were performed:

1. The 5′ and 3′ flank coordinates of each circRNA found in sheep were converted to human coordinates with the USCS liftOver tool^29^ with default parameters (min. ratio of remapped bases = 0.95).
2. The resulting coordinates were screened for overlap with human annotated circRNAs in CIRCpedia. Splice sites detected in ± 2 nt intervals around the putative human sites were considered homologous.
3. Different categories were assigned to each circRNA: “not-aligned”, the sheep coordinates were not translated to human with liftOver; “no homologous”, no human circRNA detected near both splice sites; “5′ site utilized”, a human circRNA that only uses the 5′ splice site is detected; “3′ site utilized”, a human circRNA that only uses the 3′ splice site is detected; “Both sites utilized”, both splice sites are used by different circRNAs in human; and “homologous”, a human circRNA using both splice sites is detected.

### Enrichment analysis

The detected circRNAs whose origin was in an annotated gene were further analysed as follows. Gene enrichment analysis was conducted using the GO^31^ and KEGG^32^ databases in g:Profiler^30^. This tool computes p-values for enriched terms using a Fisher’s exact test and applies the Benjamini–Hochberg multiple test correction. The set of all expressed genes detected in the total RNA-seq libraries was set as background and related terms associated with the host genes of the circRNAs were tested for enrichment. Terms composed of more than 400 genes, due to limited interpretative value, or composed of less than 5 genes, due to the decrease in statistical power by multiple testing correction, were removed from the analysis. Those terms with an FDR less than 0.05 were selected for further analysis. For visualization purposes, the list of enriched GO term was further analysed with Cytoscape using EnrichmentMap and Autoannotate plugins^57^. EnrichmentMap generates a network in which pathways are visualized as nodes connected between each other if they share many genes. Pathways with common genes often represent similar biological processes and are grouped together as sub-networks. Clusters with less than 3 interconnected nodes were removed for visualization purposes.

### circRNAs acting as miRNA sponges

A list of predicted clusters of miRNA binding sites previously reported in the human genome (hg19) was downloaded from Pan et al*.*^33^. The genomic coordinates of each sponge candidate were converted to hg38 with liftOver (min. ratio of remapped bases = 0.95) and intersected with those of the circRNAs identified in this study, already lifted from the sheep reference genome to the human genome hg38 as explained above, with bedtools (min. fraction overlap = 75%). Results were then filtered by excluding sponges targeting miRNAs for which no high confidence orthologue sequence was reported in sheep according to Ensembl^58^ (release 97). All human miRNAs hairpins were screened for similarity with the Oar3.1 genome with BLAST, requiring a minimum sequence identity of 90% on at least 95% of the hairpin. The sequences of the processed miRNAs were downloaded from miRBase^59^ (Release 22.1) and the corresponding sheep orthologous were extracted from the alignment provided by Ensembl. CircRNAs were screened for miRNA binding sites with RIsearch2^34^, using the following parameters: -s 1:8/6 -e -10 -l 20 -p2. In the same way we re-evaluated the clusters of miRNA binding sites identified in human and noticed almost no difference compared to the binding sites previously reported (Supplementary Table S2). The same criteria were applied to find binding sites of miR-671 on the human CDR1-AS and on the corresponding sheep circRNA4960.

### Differential expression analysis

For the encephalon samples, the differential expression analysis was performed via two different methods. First, the analysis was done with DESeq2^37^, setting an adjusted p-value < 0.05 as significance cut-off. An alternative method was also applied, given that DESeq2 is not designed to work on circRNA expression data. In this case, for normalization of the circRNA expression data, not only the circRNA counts were taken into consideration to calculate library sizes, but the total amount of reads aligned to the reference annotation was considered. The data was then normalized by SRPBM (Spliced Reads per Billion Mapped Reads)^5^. After normalization, a Kruskal–Wallis test was employed to check for differences between groups, and the resulting p-values were adjusted for multiple comparisons with the Benjamini and Hochberg method. An adjusted p-value < 0.05 was taken as significance cut-off to identify the differentially expressed circRNAs.

For the PBMC samples, a batch effect removal program, harman [v1.12.0]^38^, was applied after normalizing data by SRPBM. Then, the package limma^39^ and the Kruskal–Wallis test were applied to check for differential expression. Those circRNAs with an adjusted p-value < 0.05 were taken as cut-off.

## Supplementary Information

Supplementary Data S1.

Supplementary Data S2.

Supplementary Table S1.

Supplementary Table S2.

Supplementary Table S3.

Supplementary Information.

## Data Availability

RNA-seq data have been deposited in the NCBI Gene Expression Omnibus (GEO) database with experiment accession number GSE128597 for encephalon samples and GSE113899 for PBMCs samples.

## References

[1] Guo JU, Agarwal V, Guo H, Bartel DP (2014). Expanded identification and characterization of mammalian circular RNAs. Genome Biol..

[2] Bonizzato A, Gaffo E, Te Kronnie G, Bortoluzzi S (2016). CircRNAs in hematopoiesis and hematological malignancies. Blood Cancer J..

[3] Sanger HL, Klotz G, Riesner D, Gross HJ, Kleinschmidt AK (1976). Viroids are single stranded covalently closed circular RNA molecules existing as highly base paired rod like structures. Proc. Natl. Acad. Sci. U.S.A..

[4] Nigro JM (1991). Scrambled exons. Cell.

[5] Burd CE (2012). Circular RNAs are abundant, conserved, and associated with ALU repeats. RNA.

[6] Wang D, Luo Y, Wang G, Yang Q (2019). Circular RNA expression profiles and bioinformatics analysis in ovarian endometriosis. Mol. Genet. Genomic Med..

[7] Sekar S, Liang WS (2019). Circular RNA expression and function in the brain. Non-coding RNA Res..

[8] Ebbesen KK, Hansen TB, Kjems J (2017). Insights into circular RNA biology. RNA Biol..

[9] Chioccarelli T (2020). Histone post-translational modifications and circRNAs in mouse and human spermatozoa: Potential epigenetic marks to assess human sperm quality. J. Clin. Med..

[10] Ragan C, Goodall GJ, Shirokikh NE, Preiss T (2019). Insights into the biogenesis and potential functions of exonic circular RNA. Sci. Rep..

[11] Legnini I (2017). Circ-ZNF609 is a circular RNA that can be translated and functions in myogenesis. Mol. Cell.

[12] Du WW (2016). Foxo3 circular RNA retards cell cycle progression via forming ternary complexes with p21 and CDK2. Nucleic Acids Res..

[13] Akhter, R. Circular RNA and Alzheimer’s disease. in *Advances in Experimental Medicine and Biology* Vol. 1087, 239–243 (Springer, New York, 2018). 10.1007/978-981-13-1426-1_19 30259371

[14] Zhang H, Jiang L-H, Sun D-W, Hou J-C, Ji Z-L (2018). CircRNA: A novel type of biomarker for cancer. Breast Cancer.

[15] Xia X, Tang X, Wang S (2019). Roles of CircRNAs in autoimmune diseases. Front. Immunol..

[16] Khan Z (2013). Slow CCL2-dependent translocation of biopersistent particles from muscle to brain. BMC Med..

[17] Gherardi RK, Eidi H, Crépeaux G, Authier FJ, Cadusseau J (2015). Biopersistence and brain translocation of aluminum adjuvants of vaccines. Front. Neurol..

[18] Crépeaux G (2015). Highly delayed systemic translocation of aluminum-based adjuvant in CD1 mice following intramuscular injections. J. Inorg. Biochem..

[19] WHO (2008). WHO Statement from the Global Advisory Committee on Vaccine Safety on aluminium-containing vaccines.

[20] Tomljenovic L, Shaw CA (2011). Aluminum vaccine adjuvants: Are they safe?. Curr. Med. Chem..

[21] Luján L (2013). Autoimmune/autoinflammatory syndrome induced by adjuvants (ASIA syndrome) in commercial sheep. Immunol. Res..

[22] Varela-Martínez E (2018). Molecular signature of aluminum hydroxide adjuvant in ovine PBMCs by integrated mRNA and microRNA transcriptome sequencing. Front. Immunol..

[23] Varela-Martínez E (2020). Whole transcriptome approach to evaluate the effect of aluminium hydroxide in ovine encephalon. Sci. Rep..

[24] Hoffmann S (2014). A multi-split mapping algorithm for circular RNA, splicing, trans-splicing and fusion detection. Genome Biol..

[25] Cheng J, Metge F, Dieterich C (2016). Specific identification and quantification of circular RNAs from sequencing data. Bioinformatics.

[26] Holdt LM, Kohlmaier A, Teupser D (2018). Molecular roles and function of circular RNAs in eukaryotic cells. Cell. Mol. Life Sci..

[27] Hanan M, Soreq H, Kadener S (2017). CircRNAs in the brain. RNA Biol..

[28] Dong R, Ma XK, Li GW, Yang L (2018). CIRCpedia v2: An updated database for comprehensive circular RNA annotation and expression comparison. Genomics Proteomics Bioinform..

[29] Haeussler M (2019). The UCSC genome browser database: 2019 update. Nucleic Acids Res..

[30] Raudvere U (2019). g:Profiler: A web server for functional enrichment analysis and conversions of gene lists (2019 update). Nucleic Acids Res..

[31] Ashburner M (2000). Gene ontology: Tool for the unification of biology. Nat. Genet..

[32] Kanehisa M (2000). KEGG: Kyoto Encyclopedia of Genes and Genomes. Nucleic Acids Res..

[33] Pan X, Wenzel A, Jensen LJ, Gorodkin J (2018). Genome-wide identification of clusters of predicted microRNA binding sites as microRNA sponge candidates. PLoS ONE.

[34] Alkan F (2017). RIsearch2: Suffix array-based large-scale prediction of RNA–RNA interactions and siRNA off-targets. Nucleic Acids Res..

[35] Haddad G, Lorenzen JM (2019). Biogenesis and function of circular RNAs in health and in disease. Front. Pharmacol..

[36] Piwecka M (2017). Loss of a mammalian circular RNA locus causes miRNA deregulation and affects brain function. Science.

[37] Love MI (2014). Moderated estimation of fold change and dispersion for RNA-seq data with DESeq2. Genome Biol..

[38] Oytam Y (2016). Risk-conscious correction of batch effects: Maximising information extraction from high-throughput genomic datasets. BMC Bioinform..

[39] Ritchie ME (2015). Limma powers differential expression analyses for RNA-sequencing and microarray studies. Nucleic Acids Res..

[40] Shan C (2019). Biogenesis, functions and clinical significance of circRNAs in gastric cancer. Mol. Cancer.

[41] Rybak-Wolf A (2014). Circular RNAs in the mammalian brain are highly abundant, conserved, and dynamically expressed. Mol. Cell.

[42] Barrett SP, Salzman J (2016). Circular RNAs: Analysis, expression and potential functions. Development.

[43] Yu CY, Kuo HC (2019). The emerging roles and functions of circular RNAs and their generation. J. Biomed. Sci..

[44] Qian Z (2018). Potential diagnostic power of blood circular RNA expression in active pulmonary tuberculosis. EBioMedicine.

[45] Li C (2017). Genome-wide analysis of circular RNAs in prenatal and postnatal pituitary glands of sheep. Sci. Rep..

[46] Li X (2019). Comprehensive expression profiling analysis of pituitary indicates that circRNA participates in the regulation of sheep estrus. Genes (Basel).

[47] Li C (2017). Genome-wide analysis of circular RNAs in prenatal and postnatal muscle of sheep. Oncotarget.

[48] Cao Y (2018). Expression profiles of circular RNAs in sheep skeletal muscle. Asian-Australas. J. Anim. Sci..

[49] Tang B, Hao Z, Zhu Y, Zhang H, Li G (2018). Genome-wide identification and functional analysis of circRNAs in *Zea**mays*. PLoS ONE.

[50] Dai X (2018). RNA-binding protein trinucleotide repeat-containing 6A regulates the formation of circular RNA circ0006916, with important functions in lung cancer cells. Carcinogenesis.

[51] Zhu L, Liu Y, Yang Y, Mao XM, Yin ZD (2019). CircRNA ZNF609 promotes growth and metastasis of nasopharyngeal carcinoma by competing with microRNA-150-5p. Eur. Rev. Med. Pharmacol. Sci..

[52] Ashwal-Fluss R (2014). CircRNA biogenesis competes with pre-mRNA splicing. Mol. Cell.

[53] Su M (2019). Circular RNAs in cancer: Emerging functions in hallmarks, stemness, resistance and roles as potential biomarkers. Mol. Cancer.

[54] Bolger AM, Lohse M, Usadel B (2014). Trimmomatic: A flexible trimmer for Illumina sequence data. Bioinformatics.

[55] Kim D, Langmead B, Salzberg SL (2015). HISAT: A fast spliced aligner with low memory requirements. Nat. Methods.

[56] Dobin A (2013). STAR: Ultrafast universal RNA-seq aligner. Bioinformatics.

[57] Reimand J (2019). Pathway enrichment analysis and visualization of omics data using g:Profiler, GSEA, Cytoscape and EnrichmentMap. Nat. Protoc..

[58] Zerbino DR (2018). Ensembl 2018. Nucleic Acids Res..

[59] Kozomara A, Birgaoanu M, Griffiths-Jones S (2019). MiRBase: From microRNA sequences to function. Nucleic Acids Res..

