# Supplementary material for: Novel circRNA discovery in sheep shows evidence of high backsplice junction conservation
Source: Sci Rep. 2021 Jan 11;11:427. doi: 10.1038/s41598-020-79781-2 (PMC7801505; doi:10.1038/s41598-020-79781-2)
Supplement: Supplementary file 6 — Supplementary Information. [file 41598_2020_79781_MOESM6_ESM.docx]

**Novel circRNA discovery in sheep shows evidence of high backsplice junction conservation.**

**Endika Varela-Martínez^1^, Giulia I. Corsi^2^, Christian Anthon^2^, Jan Gorodkin^2^* & Begoña M. Jugo^1^***

^1^Department of Genetics, Physical Anthropology and Animal Physiology, Faculty of Science and Technology, University of the Basque Country (UPV/EHU), Bº Sarriena, Leioa, 48940, Spain.

^2^Center for Non-Coding RNA in Technology and Health, Department of Veterinary and Animal Sciences, University of Copenhagen, Thorvaldsensvej 57, 1871 Frederiksberg, Denmark.

*Correspondence and requests for materials should be addressed to B.M.J. (email: begonamarina.jugo@ehu.eus) or J.G. (email: gorodkin@rth.dk).

# Supplementary material


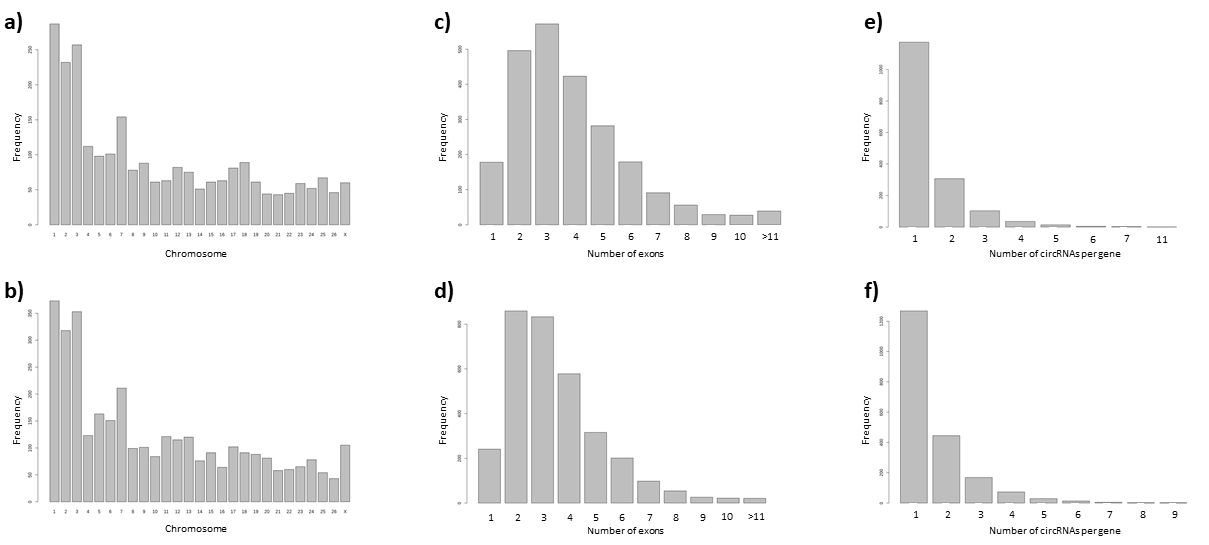


**Supplementary Figure S1:** Plots depicting some characteristic circRNA properties. a) and b) Number of circRNAs identified in each chromosome in encephalon and PBMCs, respectively. c) and d) Number of exons inside each circRNA whose origin is an annotated gene in encephalon and PBMCs, respectively. On the x-axis the number of exons a circRNA has from start-end coordinates and on the y-axis the number of circRNAs that are composed of a determined number of exons. e) and f) Bar plots in which the x-axis represents how many circRNAs are from the same host gene and the y-axis shows the number of genes that host a specific number of circRNAs in encephalon and PBMCs, respectively.


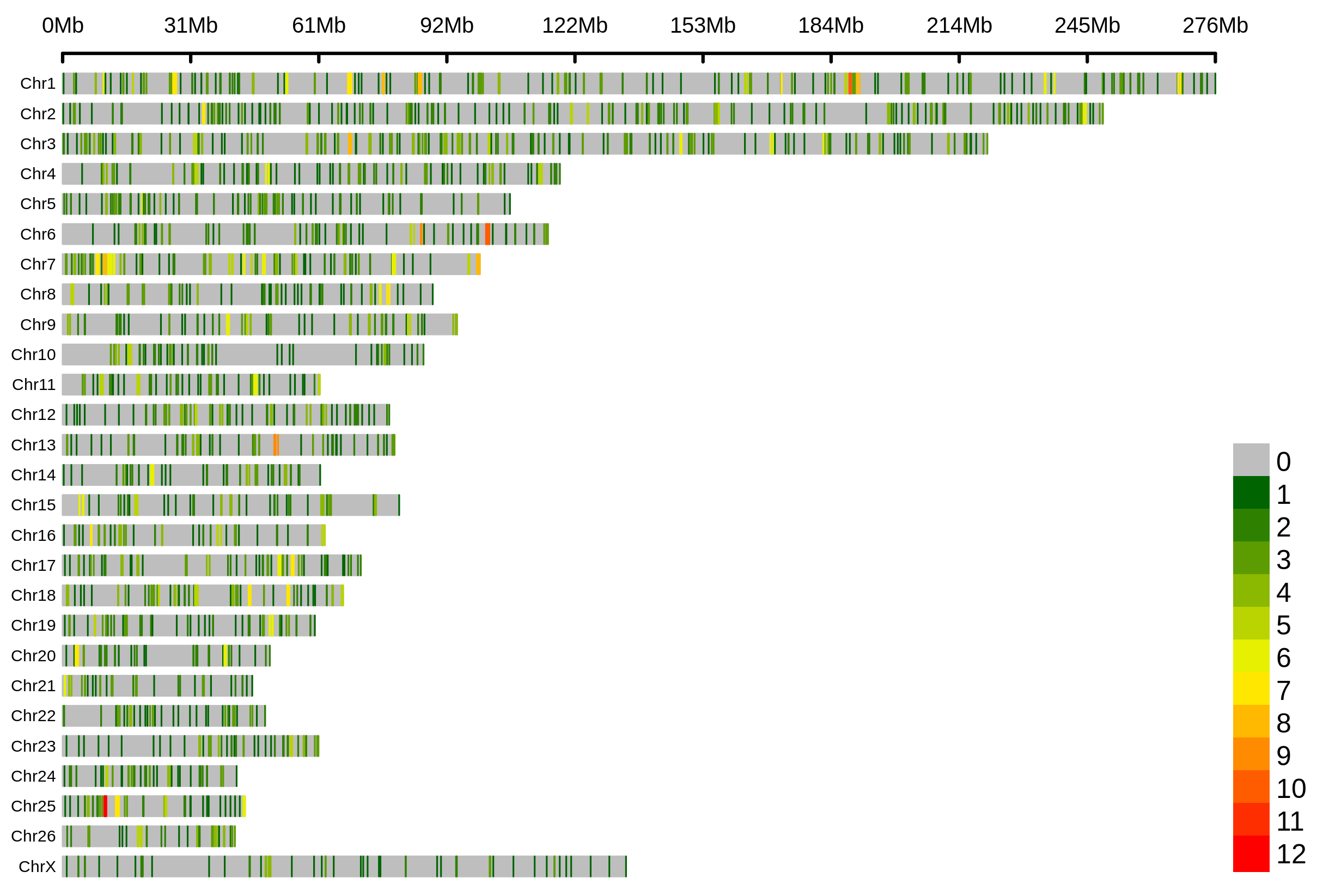


**Supplementary Figure S2:** Location of detected circRNAs in encephalon. Each chromosome was divided in bins of 1Mb and the number of circRNAs was counted in each bin. The colour code represents the number of circRNAs detected in the bins.


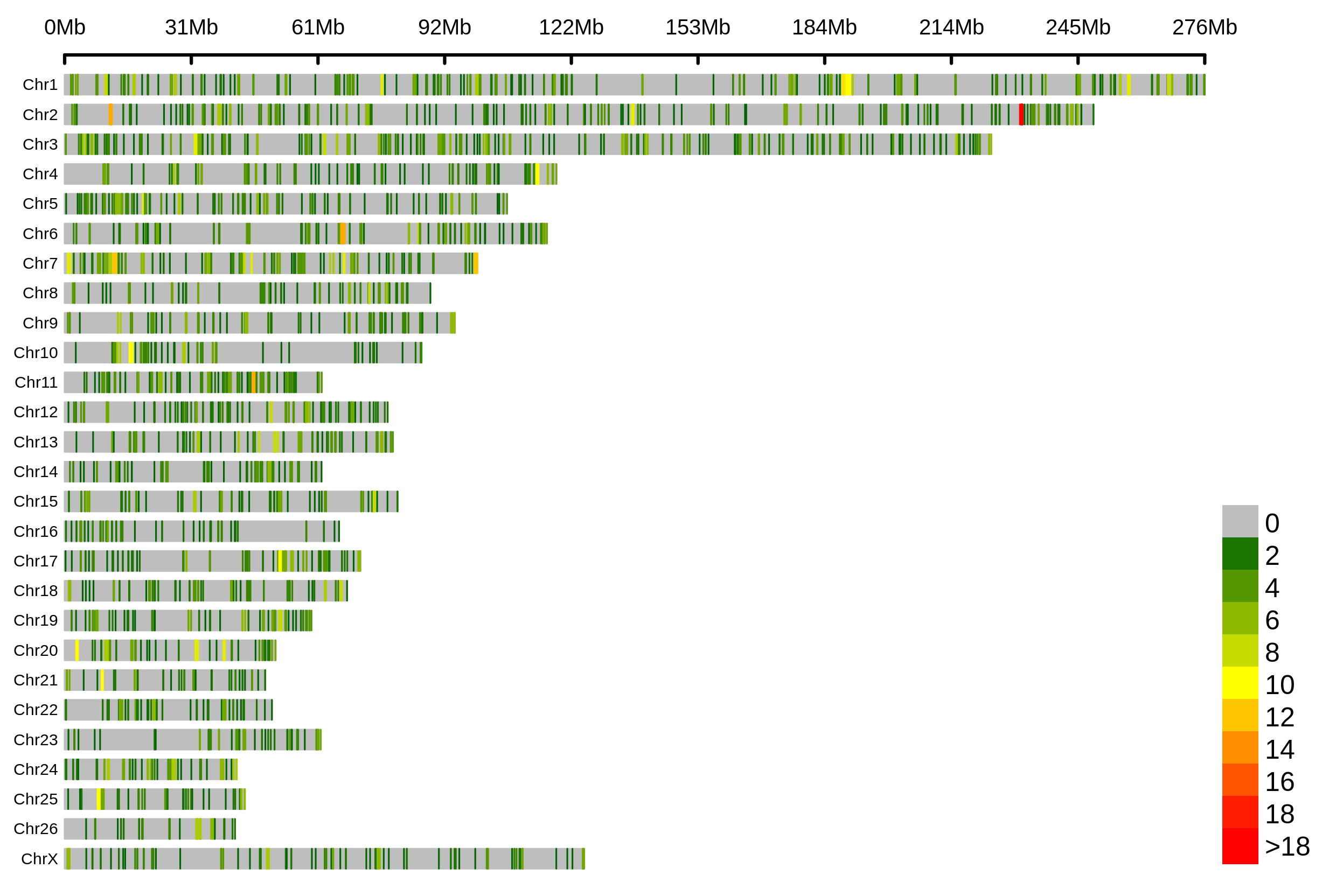


**Supplementary Figure S3:** Location of detected circRNAs in PBMCs. Each chromosome was divided in bins of 1Mb and the number of circRNAs was counted in each bin. The colour code represents the number of circRNAs detected in the bins.

**Supplementary Figure S4** Complete network from enriched GO terms by g:Profiler in encephalon and visualized in Cytoscape after clustering with Autoannotate. Node size correspond to number of genes expressed from the term; edge size represents the number of genes that overlap between different terms; and colour represents the significance level (FDR).

**Supplementary Figure S5:** Complete network from enriched GO terms by g:Profiler in PBMCs and visualized in Cytoscape after clustering with Autoannotate. Node size correspond to number of genes expressed from the term; edge size represents the number of genes that overlap between different terms; and colour represents the significance level (FDR).


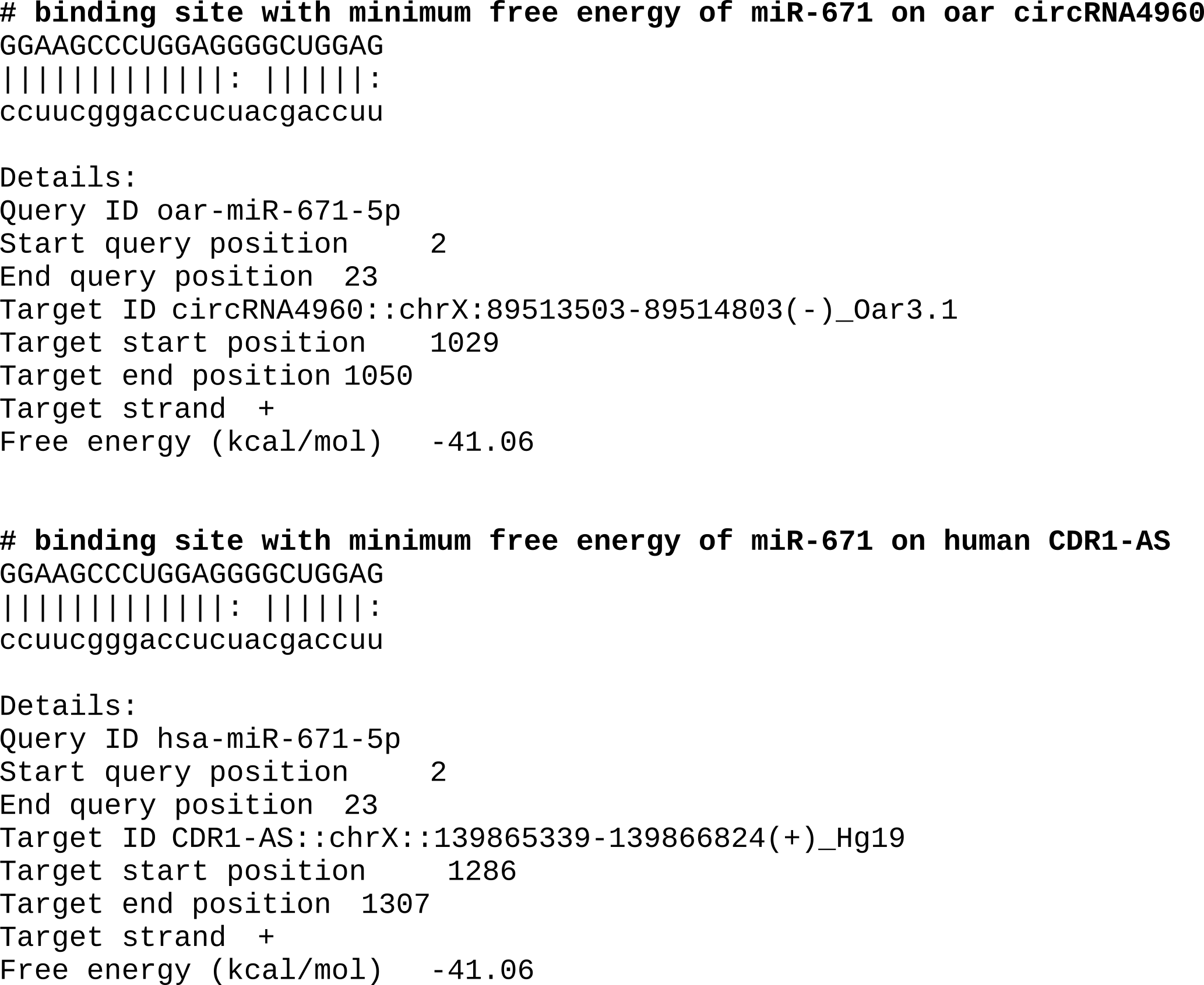
**Supplementary Figure S6:** binding patterns with minimum free energy of miR-671 on sheep circRNA4960 and human CDR1-AS, computed with Risearch2 (see Materials and Methods).


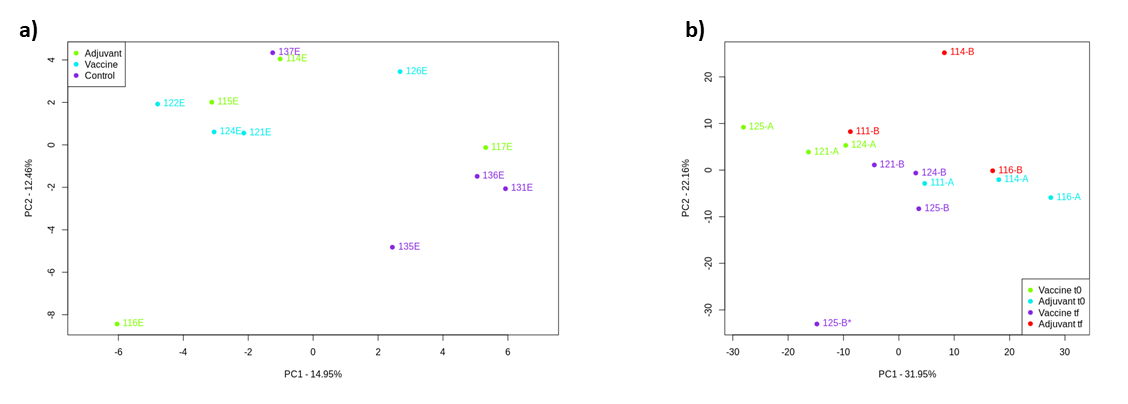


**Supplementary Figure S7:** Principal component analysis (PCA) of the encephalon and PBMCs circRNA data. a) PCA plot for the encephalon samples. b) PCA plot for the PBMC samples after batch effect correction with the Harman package from R.

**Supplementary Table S1:** circRNAs in sheep with no related gene in the annotations, but homologous to a human circRNA whose origin is a known human gene. The column CIRCpedia marks which is the human homologous circRNA, while the Gene column marks to which gene the circRNA is related in human.

**Supplementary Table S2:** Candidate sponges and corresponding miRNA targets that are common between human (clusters of miRNA binding sites) and sheep (circRNAs). Entries in bold represent circRNAs whose target miRNA has an orthologue in sheep. The lists of miRNA binding sites for each miRNAs-circRNA pair are also included.

**Supplementary Table S3:** Top highly expressed circRNAs in encephalon and PBMCs. From each sample the 10 most expressed circRNAs where extracted. The column “Sample” describes in how many samples the circRNA has been detected as top 10.

**Supplementary Data S1:** Information on the filtered encephalon circRNAs, with Oar3.1 as the reference genome. Chr: Chromosome. Start: First coordinate of the backsplice junction. End: Second coordinate of the backsplice junction. CIRCpedia: To which human circRNA is homologous. Human_Gene: The human gene that originates the corresponding human circRNA.

**Supplementary Data S2:** Information on the filtered PBMC circRNAs, with Oar3.1 as the reference genome. Chr: Chromosome. Start: First coordinate of the backsplice junction. End: Second coordinate of the backsplice junction. CIRCpedia: To which human circRNA is homologous. Human_Gene: The human gene that originates the corresponding human circRNA.
